# Effect of individually tailored biopsychosocial workplace interventions on chronic musculoskeletal pain, stress and work ability among laboratory technicians: randomized controlled trial protocol

**DOI:** 10.1186/1471-2474-15-444

**Published:** 2014-12-18

**Authors:** Kenneth Jay, Mikkel Brandt, Emil Sundstrup, MC schraefel, Markus D Jakobsen, Gisela Sjøgaard, Lars L Andersen

**Affiliations:** National Research Centre for the Working Environment, Lersø Parkallé, 105 Copenhagen, Denmark; Department of Sports Science and Clinical Biomechanics, University of Southern Denmark, Odense, Denmark; Electronics and Computer Science, University of Southampton, Southampton, UK; Center for Sensory-Motor Interaction (SMI), Department of Health Science and Technology, Aalborg University, Denmark; Royal Academy of Engineering Research Chair, London, UK; Engineering and Physical Sciences Research Council, Swindon, UK

**Keywords:** Musculoskeletal disorders, Occupational health and performance, Neck pain, Shoulder pain, Elbow pain, Hand pain, Wrist pain, Repetitive work, Stress, Work ability

## Abstract

**Background:**

Among laboratory technicians, the prevalence of neck and shoulder pain is widespread possibly due to typical daily work tasks such as pipetting, preparing vial samples for analysis, and data processing on a computer including mouse work - all tasks that require precision in motor control and may result in extended periods of time spent in static positions.

In populations characterized by intense chronic musculoskeletal pain and diagnosed conditions in conjunction with psycho-physiological symptoms such as stress-related pain and soreness and other disabling conditions, multifactorial approaches applying a combination of individually tailored physical and cognitive strategies targeting the areas most needed, may be an effective solution to the physical and mental health challenges.

The aim of this study is therefore to investigate the effect of an individually tailored biopsychosocial intervention strategy on musculoskeletal pain, stress and work disability in lab technicians with a history of musculoskeletal pain at a single worksite in Denmark.

**Methods/design:**

In this single-blind two-armed parallel-group randomized controlled trial with allocation concealment, participants receive either an individualized multifactorial intervention or “usual care” for 10 weeks at the worksite. Inclusion criteria: 1) female laboratory technician (18-67 years of age) and 2) Pain intensity ≥ 3 (0-10 Visual Analogue Scale) lasting ≥3 months with a frequency of ≥ 3 days per week in one or more of the following regions: i) upper back i) low back iii) neck, iv) shoulder, v) elbow and/or vi) hand. Exclusion criteria: 1) life-threatening disease and 2) pregnancy. Stress, as measured by Cohen´s perceived stress questionnaire is not an inclusion criteria, thus participants can participate regardless of their stress level.

We will implement an individualized intervention addressing biopsychosocial elements of musculoskeletal pain with the following components; i) increasing physical capacity through strength- and motor control training; ii) lowering or preventing development of stress through mindfulness practice and learning de-catastrophizing pain management strategies through cognitive training.

The primary outcome at 10-week follow-up is the between-group difference in intensity of perceived musculoskeletal pain during the last week (average value of back, neck, shoulder, elbow and hand) assessed by questionnaire (modified visual analogue scale 0-10).

**Discussion:**

This study will provide experimental evidence to guide workplace initiatives designed towards reducing chronic musculoskeletal pain and stress.

**Trial registration number:**

ClinicalTrials.gov NCT02047669.

**Electronic supplementary material:**

The online version of this article (doi:10.1186/1471-2474-15-444) contains supplementary material, which is available to authorized users.

## Background

Musculoskeletal disorders comprise a major socioeconomic burden on public health systems in North America and Europe [1]. Pain in the upper extremity accounts for the majority (20-30%) of health complaints in the adult working population [2, 3]. Repetitive movement tasks with static contractions are unavoidable in many occupations and have been associated with musculoskeletal pain and myalgias [2, 4, 5]. Neck and shoulder pain have been a primary outcome in several investigations examining the effects of physical exercise on musculoskeletal pain in the upper extremity [6–9]. In occupational groups with a high prevalence of pain in the neck and upper extremity [10, 11], strength training appears to be a clinically sound approach to implement at the worksite [12, 13]. In other occupational environments such as among laboratory technicians, the prevalence of neck and shoulder pain is also widespread due to typical daily work tasks such as pipetting, preparing vial samples for analysis, and data processing on a computer including mouse work - all tasks that require precision in motor control and may result in extended periods of time spent in static working postures [14, 15]. For laboratory technicians there are positive outcomes from a 20 week RCT with high-intensity strength training, relying on principles of progressive overload, for reducing the intensity of non-chronic musculoskeletal pain of the neck and shoulder [4]. However, when dealing with populations suffering from more intense chronic (lasting more than 3 months) musculoskeletal pain in conjunction with psycho-physiological symptoms such as stress-related pain and soreness, lack of concentration ability, insomnia and other disabling conditions, strategies beside high-intensity strength training may be more effective in reducing pain. One interesting multifactorial approach is to perform individual needs analyses and subsequently applying a combination of physical and cognitive strategies individually tailored in such a way, that the participant would be offered targeted training and rehabilitation in the area most needed.

### Physical activity

Our research group has previously shown clinically relevant reductions in back, neck, shoulder and arm pain in response to 10–20 weeks of strength training using kettlebells [16, 17], elastic resistance bands [18, 19] or free weight exercises [5, 12, 13] in office workers and laboratory technicians, which supports the positive effect of simple strength training in people with musculoskeletal pain [4]. However, although strength training reduced the intensity of musculoskeletal pain with moderate to large effect sizes subjects did typically not become pain-free. Furthermore, these studies excluded employees with specific musculoskeletal diagnoses, e.g. impingement, radicular pain or carpal tunnel syndrome. Such a strategy may exclude those most in need of rehabilitation. From a theoretical point of view, increasing the physical capacity of laboratory technicians could reduce the relative load placed upon them during work and thereby reduce the musculoskeletal strain but in contrast, targeted strength training interventions may not be feasible in people suffering from clinically diagnosed conditions such as carpal tunnel syndrome or other severe conditions. In such cases, a different approach may be necessary, as typical progressive strength training protocols can be speculated to be too strenuous and thereby not be a viable treatment method. Alternatively, it might be more beneficial for some individuals to drive the intervention strategy towards basic joint mobility and the practice of precise motor control in close combination with targeted drills to relieve pain symptoms.

### Fear avoidance

While a model that focuses upon structural and biomechanical abnormalities may help explain and alleviate musculoskeletal pain it cannot sufficiently explain more severe states of chronic pain and their associated disability.

The International Association for the Study of Pain (IASP) defines pain as: “*An unpleasant sensory and emotional experience associated with actual or potential tissue damage, or described in terms of such damage*”, which has brought attention towards fear as a psychological factor greatly influencing chronic pain [20]. The literature shows a host of physiological mechanisms by which injuries lead to nociceptive responses and ultimately to pain but not all nociceptive signals are perceived as pain and not every pain sensation originates from nociception [21–23]. This point towards a centrally governed control mechanism that ultimately determines whether a stimulus is perceived as painful. Psychological research on the effect of fear and anxiety on chronic pain has been extensively recognised. Pain-related fear and anxiety can best be defined as the fear that emerges when stimuli that are related to pain are perceived as a threat [24]. The fear and anxiety response comprises psycho-physiological (e.g. increased muscle tension), behavioural (e.g. escape and avoidance behaviour), as well as cognitive (e.g. catastrophizing thoughts) elements. Thus, the fear of pain, fear of work-related activities, fear of movement, and fear of (re)injury have been described as often occurring in patients suffering from chronic pain [24]. In such cases, learning de-catastrophizing coping strategies through cognitive behavioural training and re-educating the body-self neuromatrix [21, 22, 25, 26] may serve as valuable intervention strategies to implement with people showing movement related fear-avoidance behaviour [27, 28].

### Fatigue and stress

There is evidence supportive of the relationship between musculoskeletal pain and fatigue. For instance, chronic pain accounts for up to 34% of self-reported activity limitations in patients suffering from Chronic Fatigue Syndrome (CFS) [29] and it is suggested that myalgia and arthralgia could be considered an important subclass of CFS [30]. The research in CFS shows that widespread and consistent pain is common. For instance, a population-based study revealed that 94% of the persons diagnosed with CFS reported muscle aches and pain and 84% reported joint pain [31]. CFS patients reported having muscle pain in 85 out of 114 instances and 74 patients complained of arthralgia [32]. Women with trapezius myalgia also suffer higher fatigue levels in the trapezius muscle compared to healthy controls even at rest [33]. Given the amount of convincing data on the relationship between severe chronic fatigue and musculoskeletal pain [34] it may be appropriate to fit fatigue as an element of chronic musculoskeletal pain in a biopsychosocial intervention strategy.

The link between musculoskeletal pain and situational stress should also be taken into account [35]. Stress can have widespread effects on emotional, physical, cognitive and behavioural wellbeing. Typical symptoms of stress include frustration, fatigue, headaches, chest pain and rapid heartbeat, forgetfulness, disorganization, and tense muscles [36–38]. Importantly, a recent prospective cohort study showed that high muscle tension was associated with almost 4-fold increased chance of developing neck pain 1 year later [39]. The symptoms of stress are similar to how CFS is defined by the Centers for Disease Control and Prevention (CDCP), who defines it as *“a complex illness characterized by prolonged debilitating fatigue and multiple non-specific symptoms including headaches, re- current sore throats, fever, muscle and joint pain, and neurocognitive complaints”*[34]. Based on psychological research a biopsychosocial intervention strategy aimed at reducing musculoskeletal pain by taking fatigue and situational stress into account seems appropriate to investigate, as psychosomatic research shows an isomorphic- (same time increases in pain lead to same time increases in stress and vice versa), consequence- (increases in pain precede increases in stress), and a precursor (increases in stress precede increases in pain) stress-pain relationship [35]. This supports a psychological theory of using individually tailored multifactorial interventions in chronic pain rehabilitation settings.

The aim of this study is therefore to investigate the effect of an individually tailored biopsychosocial intervention strategy versus company policy ergonomics and on-going exercise initiatives on chronic musculoskeletal pain, stress and work disability in lab technicians with a history of work-related musculoskeletal pain.

## Methods

### Trial design

This trial follows a single-blind randomized controlled design with allocation concealment in a two-armed parallel group format among laboratory technicians at a single worksite with multiple departments in Denmark. The participants are parallel-assigned to receive either physical-cognitive-mindfulness training or follow company policies for 10 weeks at the worksite. The study duration is March 2014 (baseline testing) to July 2014 (follow-up testing) (with 1-year follow-up scheduled for March 2015).

### Ethics

Ethical approval has been obtained from The Danish National Committee on Biomedical Research Ethics (The local ethical committee of Frederiksberg and Copenhagen; H-3-2010-062) as part of the research program “Implementation of physical exercise at the workplace (IRMA)”. The trial *“Implementation of physical exercise at the Workplace (IRMA09) – Laboratory technicians”* is registered in the ClinicalTrials.gov register (NCT02047669) prior participant enrolment.

### Participant recruitment

A recruitment questionnaire on musculoskeletal pain, stress and work disability are sent out to 752 laboratory technicians in a division of a large pharmaceutical company in Denmark. Based on previous work [13, 40] we conservatively estimate a recruitment level of 10-15% or 75-110 participants with chronic musculoskeletal pain. The participants must be, at the time of enrolment, suffering from chronic musculoskeletal pain in one or more of the following regions: i) upper back, ii) lower back iii) neck, iv) shoulders, v) elbows or vi) hands/wrists to participate in the study. To fulfil the definition of chronic pain all following criteria must be met for at least one of these body regions: i) pain intensity of ≥3 (0-10 Visual Analogue Scale) during the last week, ii) pain frequency of ≥3 days during the last week, iii) the pain should have lasted at least 3 months.

We will not exclude participants due to disease unless contraindications for all elements of the intervention exist. Instead, participants with typical exclusion criteria, e.g. severe hypertension, are allowed to participate in the less strenuous part of the intervention if their own doctor clears them. Thus, special emphasis of the individually tailored intervention will be to offer interventions for all employees with chronic musculoskeletal pain unless contraindications exist. Life-threatening disease and pregnancy are considered contraindications to the testing and training and we will therefore exclude such participants. Finally, stress, as measured by Cohen´s perceived stress questionnaire is not an inclusion criteria, thus participants can participate regardless of their stress level.

We will describe baseline demographics and important variables in the main article reporting results on the primary outcome. These include: i) age, ii) height, iii) weight, iv) BMI, v) pain intensity, vi) pain frequency, vii) pain duration since onset, and viii) prescription medicine, ix) other treatment modalities (e.g. by a doctor or physical therapist), x) Cohen’s stress score.

All participants will be informed about the purpose and content of the project and must provide their written informed consent to participate in the study. All experimental conditions conform to The Declaration of Helsinki.

### Randomization

By generation of a random numbers table in the SAS statistical software the eligible participants are allocated to either a physical-cognitive-mindfulness intervention (PCMT) or a reference group (REF) following company on-going initiatives including standard ergonomic policy. Participants are informed about their particular group allocation by email after the baseline data acquisition. Because participants cannot be blinded to group allocation we will inform them that it is unknown which treatment model works best. Participants are instructed to not reveal their particular intervention to the assessors during follow-up examination. Should the experimental treatment prove to be superior to the standard company policy the reference group is offered the experimental treatment after follow-up testing.

### Blinding

Due to the interventional trial design instructors (physical training instructors and mindfulness instructors) cannot be blinded to group allocation. Further, participants cannot be blinded to which intervention treatment they are going to receive. Outcome assessors and data analysts will be blinded to participant group allocation. Thus, the individuals performing the data collection (assessors) are not involved in the training nor the statistical analysis, and do not know which group the subjects are allocated to. The training- and mindfulness instructors are paid to conduct the teaching and are not researchers and therefore not involved in any other part of the study. The individual performing the statistical analyses are not involved in the testing or the training in any way.

### Interventions

We aim to implement an experimental approach, multifactorial and tailor-made to the individual addressing biopsychosocial elements of musculoskeletal pain by increasing the level physical activity through strength training, mobility training, and motor control training. Further, we aim to lower stress through mindfulness. Additionally, to address the psychological element of musculoskeletal pain we include de-catastrophizing pain management strategies and fear avoidance belief education Finally, we will structure the intervention so participation in group based physical and/or mental exercise sessions will increase social interaction through guided activity.

The physical-cognitive-mindfulness training intervention design (PCMT) has four elements. Each treatment modality is described in detail below.

### PCMT element 1 - strength training

**Figure 1:**
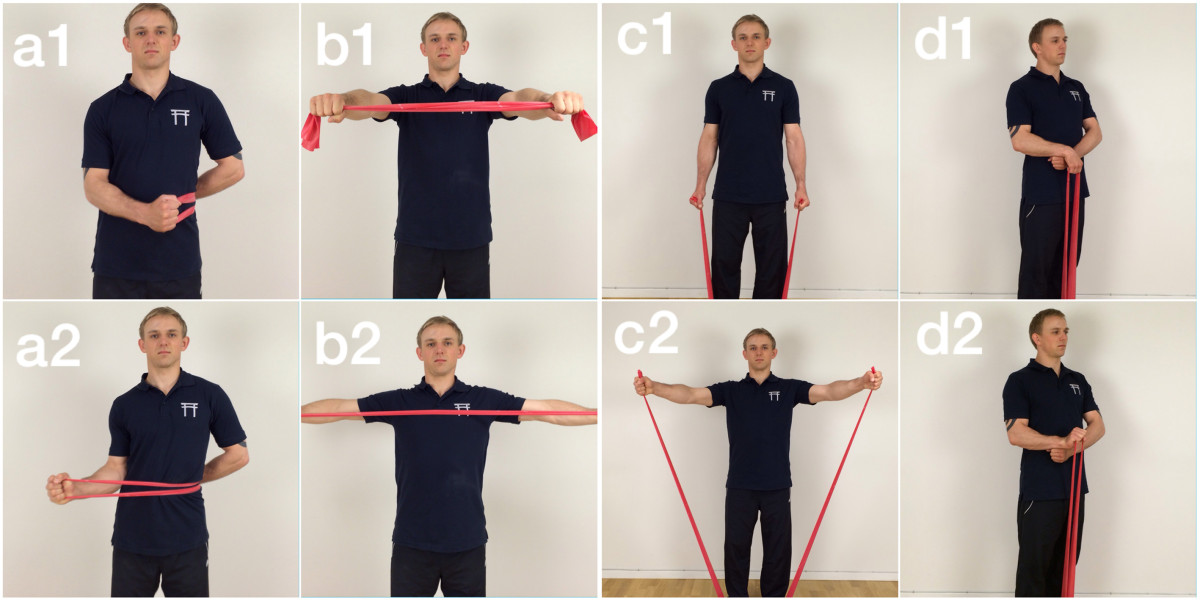
**Shows the three primary elastic tubing exercises used during the intervention.** Exercise a shows shoulder external rotation start **(a1)** and end **(a2)** and exercise b shows shoulder squeeze start **(b1)** and end **(b2)**. Finally, exercise c shows lateral raise start **(c1)** and end **(c2)** and exercise d shows wrist extension start **(d1)** and end **(d2)**.

The strength training is targeted towards the site of musculoskeletal pain. Simple elastic resistance exercises for the neck/shoulder, arm, wrist and hand are utilized. Similar types of elastic resistance exercise have previously been validated to be as effective as traditional strength training with dumbbells [41]. The program design follows a progressive training model with variable resistance and/or contraction type and speed. The supervising instructor will adjust the exercises to fit the individual. Figure 1 shows the elastic tubing exercises. 1-2 sets of 10-20 repetitions of each exercise are performed.

### PCMT element 2 - precise motor control training

**Figure 2:**
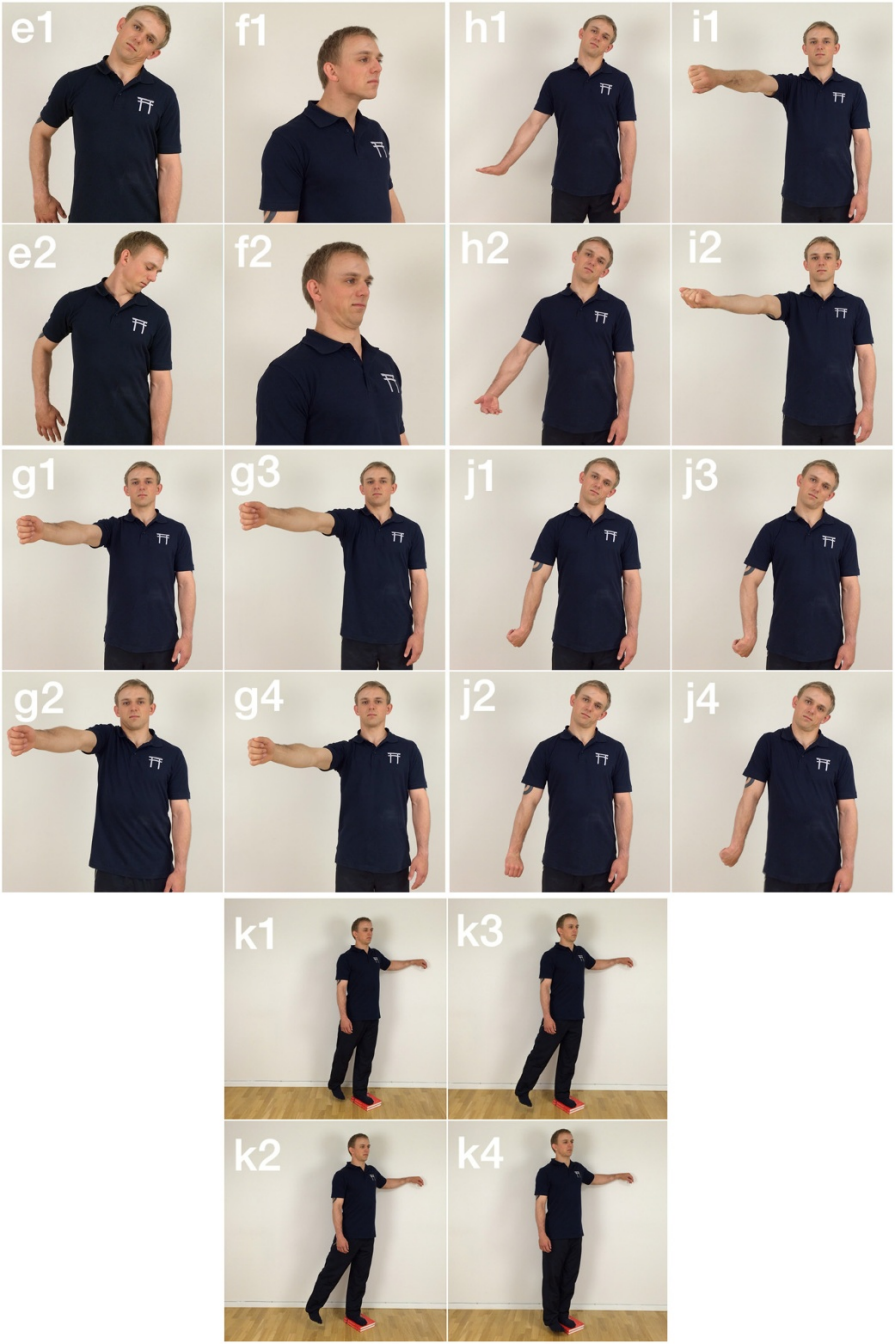
**Shows the seven primary mobility and motor control exercises used during the intervention.** Exercise e and f shows axillary mobilization start **(e1)** and end **(e2)** and cervical mobilization start **(f1)** and end **(f2)**, respectively. Exercises g, h and I show shoulder camshaft mobilization start **(g1)**, ¼ of the way **(g2)**, ¾ of the way **(g3)** and end **(g4)**, brachial external rotation mobilization start **(h1)** and end **(h2)** and shoulder internal/external distraction mobilization start **(i1)** and end **(i2)**, respectively. Exercise j show brachial internal rotation mobilization start **(j1)**, ¼ of the way **(j2)**, ¾ of the way **(j3)** and end **(j4)**. Finally exercise k shows hip circular motor control and mobilization start **(k1)**, ¼ of the way **(k2)**, ¾ of the way **(k3)** and end **(k4)**.

The motor control training is based on simple isolated dynamic joint mobility movements inspired by the precise execution of tai chi and qi-gong [42] and integrated following the principles of motor learning [43, 44]. The supervising instructor will adjust the level of difficulty as well as implement alternative exercises for other body regions to fit the individual and target the site of pain. Figure 2(e-k) shows seven key motor control movement sequences utilized. Motor control exercises e and f are applied more frequently to participants complaining primarily of neck and upper back pain. Exercises g and i are applied more frequently to participants with shoulder pain, and h and j are applied to participants with arm/elbow/hand pain. Finally, exercise k is primarily applied to participants complaining of lumbar/sacroiliac pain. 2-3 sets of 3-5 repetitions done at a “super slow” speed (15-30 sec. per repetition) are performed in each direction focusing on creating a smooth continuous motion in progressively larger circles.

### PCMT element 3 - mindfulness

Mindfulness is comprised of two separate but mutually supportive elements- a passive and an active element. The passive element is based on basic meditation techniques with a focus on becoming aware of the body. This meditation exercise, known as a *body scan*, is verbally guided by the instructor and does not involve any movement. In a meditational body scan the participants will be lying down with closed eyes. The instructor will start the guided attention at the toes and feet and work up through the legs, hips, torso, arms, hands, fingers and head and for each segment ask the participants to notice the different sensations (heat, cold, restlessness etc.) from each specific area.

The active element is comprised of attention solely on breathing. The participants will either be lying down, sitting, standing or walking outside in nature focusing on their breathing pattern. The instructor informs the participants that they may experience a wide array of different thoughts but the goal is to let these thoughts pass by, returning the attention towards their breathing.

Together, the passive and active meditation techniques comprise mindfulness and is utilized to indirectly reduce musculoskeletal pain by reducing stress and fatigue [35, 37, 45–48]. The mindfulness is done in a group-based setting.

### PCMT element 4 - cognitive and behavioural training

Educational pain management sessions will focus on re-educating the body-self neuromatrix of the participants by affecting the neurosignature responsible for generating the pain. As pain is multidimensional and related to previous experience with pain, the management sessions will focus on understanding patterns of behaviour that may trigger pain in the individual [49, 50].

To combat fear-avoidance behaviour, education and counselling about fear of movement, the positive effects of movement as well as de-catastrophizing pain are the main focus areas [24, 51–56]. The physical training instructor will individualize the cognitive and behavioural element of the intervention and relate the counselling about fear-avoidance behaviour to the specific work tasks encountered by laboratory technicians in their daily activities as well as to their leisure time activities. The cognitive and behavioural training elements are combined with the physical training in such as way that pain de-catastrophizing counselling, as well as fear-avoidance behaviour counselling and education are integrated in the instruction and communication of the physical training by the instructor.

### PCMT - individualized treatment model

The recommended amount of each intervention element will be tailor-made to fit the individual based on the baseline questionnaire elements about pain, stress and work ability, as well as the baseline clinical examination by a physical therapist specializing in musculoskeletal pain.

Based on the screening questionnaire replies in the PCMT group, participants receive an email with information about their own “stress score” (Cohen’s Perceived stress) and body regions with chronic pain. Further, participants are informed that the physical exercise will be tailored according to their painful body regions and that they can participate in the mindfulness sessions to prevent development of stress (if stress levels are low) or reduce stress (if stress levels are high). In case of contraindications for high-intensity physical training (e.g. carpal tunnel syndrome), participants are informed that they can participate in the physical training sessions, but the exercises will be adjusted and not include strength training.

### PCMT - weekly intervention schedule

To make training easily accessible to the participants at the worksite, a weekly training structure of 20 min. supervised physical training is available four times per week (Monday, Tuesday, Thursday and Friday) with 3 session possibilities daily (10.00 am, 10.30 am and 11.00 am). Mindfulness is available once weekly (Wednesday) on two separate occasions (10.00 am and 11.00 am). All instructors are available for consultation and support via email throughout the intervention weeks and participants are encouraged to train and practice mindfulness during leisure time. Instructors take attendance to physical training- and mindfulness classes and monitors non-participation rates for each participant.

### PCMT - adjusting the strength training element

In case of acute worsening of pain or other contraindications during the time of physical training, the training instructor applies the following 4-stage model, previously described by our research team [57], to adjust the specific exercise.

*Stage 1*: Reduce loading intensity. A reduction in load (e.g. resistance of elastic tubing) should be implemented in the specific exercise that causes an increase in acute pain in the back, neck, shoulder, elbow or hand. A load reduction of up to 100% can be necessary, i.e. performing the movement without external resistance.

*Stage 2*: Reduced movement velocity. If a reduction in load fails to address the problem the movement velocity should be reduced.

*Stage 3*: Reduced range of motion (ROM). As a final action to solve the problem, the ROM should be reduced the point where pain is not worsened.

*Stage 4*: Exercise termination. If none of the above stages resolves the problem, the specific exercise will be terminated and replaced by a targeted joint mobility or motor control exercise.

Progression in strength and motor control training is done in reverse, i.e. increasing ROM, increasing movement velocity and increasing the load by applying external resistance. The progression, as well as the regression is supervised and controlled by the instructor present.

### REF group

The participants of the REF group receive an email with encouragements to participate in the company’s on-going initiatives, e.g. weekly elastic band group training sessions (only available in some departments) and are encouraged to continue to take “exercise breaks” whenever needed. As this is part of the existing and on-going program at the company, it can be considered “usual care”. No new interventions are added for the REF group.

### Compliance, adherence and dropouts

Compliance, adherence to training and dropouts will be tracked and reported in the main article on the primary outcome of pain. PCMT instructors will keep track of all participants for each training session and send out weekly information emails reminding the participants to prioritize the training and mindfulness sessions. Apart from increasing compliance, this procedure also eliminates any potential REF group participants finding their way to the training and mindfulness sessions, thus reducing the likelihood of cross contamination significantly.

### Co-interventions

Participants of both groups are recommended to continue their usual physical activities alongside the intervention. The company’s own health and safety professionals are available to provide ergonomic education in accordance with the standard company policy, which consists of ergonomic worksite observations by trained professionals and subsequent individualized recommendations on changing task-specific positions and adjusting ergonomic aids to fit each department structure. Each individual department management is responsible for prioritizing and utilizing the option of ergonomic support, thus making it voluntary. This is part of the existing company policy on ergonomic guidance and is equally available to all participants of both groups.

In this study, co-interventions are tracked as follows (i.e. asked by questionnaire at baseline and follow-up): 1) number of days using pain medication (typical over-the-counter pain medications, i.e. acetaminophen/paracetamol and NSAIDs) within the last week, 2) number of treatment sessions (e.g. by a medical doctor or physical therapist) for pain in the back, neck, shoulders, elbows or hands/wrists within the last month. The baseline information about this will be added to the article reporting the primary outcome, together with the follow-up results.

### Primary outcome measure

Our primary outcome measure is change in average pain intensity (lower back, upper back, neck, shoulders, elbows and hands/wrists) by questionnaire (scale 0-10) from baseline to week 10. The analysis will be adjusted for pain intensity at baseline. The regions of the body will be defined by drawings from the Nordic Questionnaire [58].

### Secondary outcome measure

Stress is measured from baseline to week 10 by Cohen’s perceived stress scale which is based on the answers of 10 questions each scored with the following categories: i) Never, ii) Almost never, iii) Sometimes, iv) Fairly often and v) Very often. Examples of questions include: *“In the past month, how often have you found that you could not cope with all the things you had to do?”* and *“In the past month, how often have you been able to control irritations in your life?”*[59].

### Other outcome measures

#### Work ability

Work ability is assessed by the Work Ability Index questionnaire [60, 61] from baseline to 10 week follow-up and cognitive performance is assessed by CNS Vital Signs [62] at baseline and follow-up. Electroencephalography (EEG) will be used to sample brain activity during the CNS Vital Signs test.

All outcome measures will be collected by trained clinical examiners and by questionnaire survey at baseline and after the 10-week intervention period.

#### Fear avoidance

Fear avoidance is evaluated by the Fear Avoidance Beliefs questionnaire (FABQ) by Waddell et al. [63] at baseline and follow-up. Briefly, the FABQ is a two-part questionnaire. The first part consists of five questions/statements about pain and physical activity and the second part consists of 11 questions/statements about how work affects the participants’ perception of pain. Each question is scored from 0-5 ranging from completely disagree (0) to completely agree (5).

#### Muscle function

Muscle strength, function and tenderness of the shoulder, arm, wrist/hand is assessed by maximal isometric voluntary contractions in a custom-built dynamometer (Bofors Elektronik, Karlskoga, Sweden) setting and by pressure-pain threshold testing (PPT) [64]. Also rate of force development (RFD), force steadiness (FS), force precision (FP) and fatigue (F) are measured by using custom-made MATLAB programs. The strength tests are a part of an extensive physical examination by trained (and blinded) medical professionals at baseline and follow-up. Muscle activation level is measured by surface electromyography (EMG) (Nexus Mark 10, Mindmedia, Netherlands) on the forearm extensors, shoulder external rotator (Infraspinatus Mm.) and descending part of the trapezius muscle. Further, surface EEG (Nexus Mark 10, Mindmedia, Netherlands) will measure global brain activity during pre- and post testing by using a single a single sensor placement on the forehead (Fpz) [65].

### Sample size and power

A priori power analysis based on previous measurements reveals that 27 participants of each group for 95% power, type I error probability of 5%, SD of 1.5 and a minimal relevant difference in pain intensity of 1.5 is sufficient to test the null-hypothesis of equality (alpha = 0.05, beta = 0.05). With an estimate of a 10% dropout during the intervention period, the minimal group size should be 30 (i.e. a total of 60 participants).

### Statistical analysis

All statistical analyses will be performed using the SAS statistical software for Windows (SAS Institute, Cary, NC). The change in perceived pain (0–10 scale) will be evaluated using a repeated measures linear mixed model with *group*, *time* and *group by time* interaction as independent variables. Subject is entered as a random effect. Analyses will be adjusted for baseline values. We will perform all statistical analyses in accordance with the intention-to-treat principle using the Proc Mixed procedure of SAS, which inherently accounts for missing values. An alpha level of <0.05 will be accepted as significant. Outcomes will be reported as between-group, least mean square differences and 95% confidence intervals from baseline to follow-up.

## Discussion

This study will provide evidence on the effectiveness of an individually tailored biopsychosocial intervention strategy versus company policy ergonomics and on-going exercise initiatives on chronic musculoskeletal pain, stress and work disability in lab technicians with a history of work-related musculoskeletal pain.

We prioritize a cost-efficient training program design with easy-to-use exercises and a minimal amount of necessary equipment based on the assumption that work site post-intervention implementation may have a higher success rate if the program design, including exercises, is transparent, inexpensive and easily integrated.

Ergonomic counselling aiming at reducing physical exposure to compromising body positions are considered the standard prescription/conventional approach on prevention and treatment of musculoskeletal disorders in various work environments. However, increasing employee physical and mental capacity by means of individualized strength-, mobility/motor control- and cognitive training at the work site may represent a useful approach for reducing chronic pain, stress and work disability in laboratory technicians.

### Limitations

Cross contamination can diminish between group-differences in workplace trials, and can be largely avoided by randomizing the workplace or department level. However, this also decreases statistical power due to the inflation factor associated with clustering. In the present study only we hypothesize a participation rate of 10-15% of all the employees who receive the questionnaire (i.e. those with chronic pain and willingness to participate), which minimizes the risk of cross contamination compared with all employees participating. Further, the instructors receive a list with names of participants in the intervention group and make sure that only employees on that list can participate, thus greatly reducing the risk of accidentally letting REF participants take part in the training and mindfulness sessions. Based on these lists, we will determine the degree of cross contamination from REF to PCMT.

Although a very unlikely scenario, we could potentially end up with multiple people suffering from fibromyalgia and/or rheumatoid arthritis, which could impair the randomization and intervention. However, this study involves full time employees, which make it improbable that we will see an abundance of fibromyalgia or rheumatoid arthritis cases. We will however, report any such cases in the main article containing results on the primary outcome.

The inability to blind participants to intervention treatment is not possible and also presents a limitation. Further, self-reported outcomes are a limitation as they may be influenced by placebo effects and outcome expectations. Finally, the intervention is comprised of several different elements, which makes it impossible to determine which parts of the intervention have effect or not. Thus, the present study is able to test the multifactorial ‘package’ of intervention elements. However, to provide some insight in this, we will provide exploratory dose-response analyses in PCMT between participation in each element and the effect on pain and stress.

The present study will provide documentation to better guide workplace initiatives to reduce chronic musculoskeletal pain among employees with repetitive and monotonous movement tasks of the shoulders, arms and hands/wrists, while shedding light on the association between pain, work disability and stress.

### Talent/model release

Consent for publication has been obtained from the person modelling the pictures in this article.

## Authors’ original submitted files for images

Below are the links to the authors’ original submitted files for images. Authors’ original file for figure 1 Authors’ original file for figure 2 Authors’ original file for figure 3 Authors’ original file for figure 4 Authors’ original file for figure 5 Authors’ original file for figure 6 Authors’ original file for figure 7 Authors’ original file for figure 8
