# Supplementary figures and images for: Effect of individually tailored biopsychosocial workplace interventions on chronic musculoskeletal pain, stress and work ability among laboratory technicians: randomized controlled trial protocol
Source: BMC Musculoskelet Disord. 2014 Dec 18;15:444. doi: 10.1186/1471-2474-15-444 (PMC4325961; doi:10.1186/1471-2474-15-444)

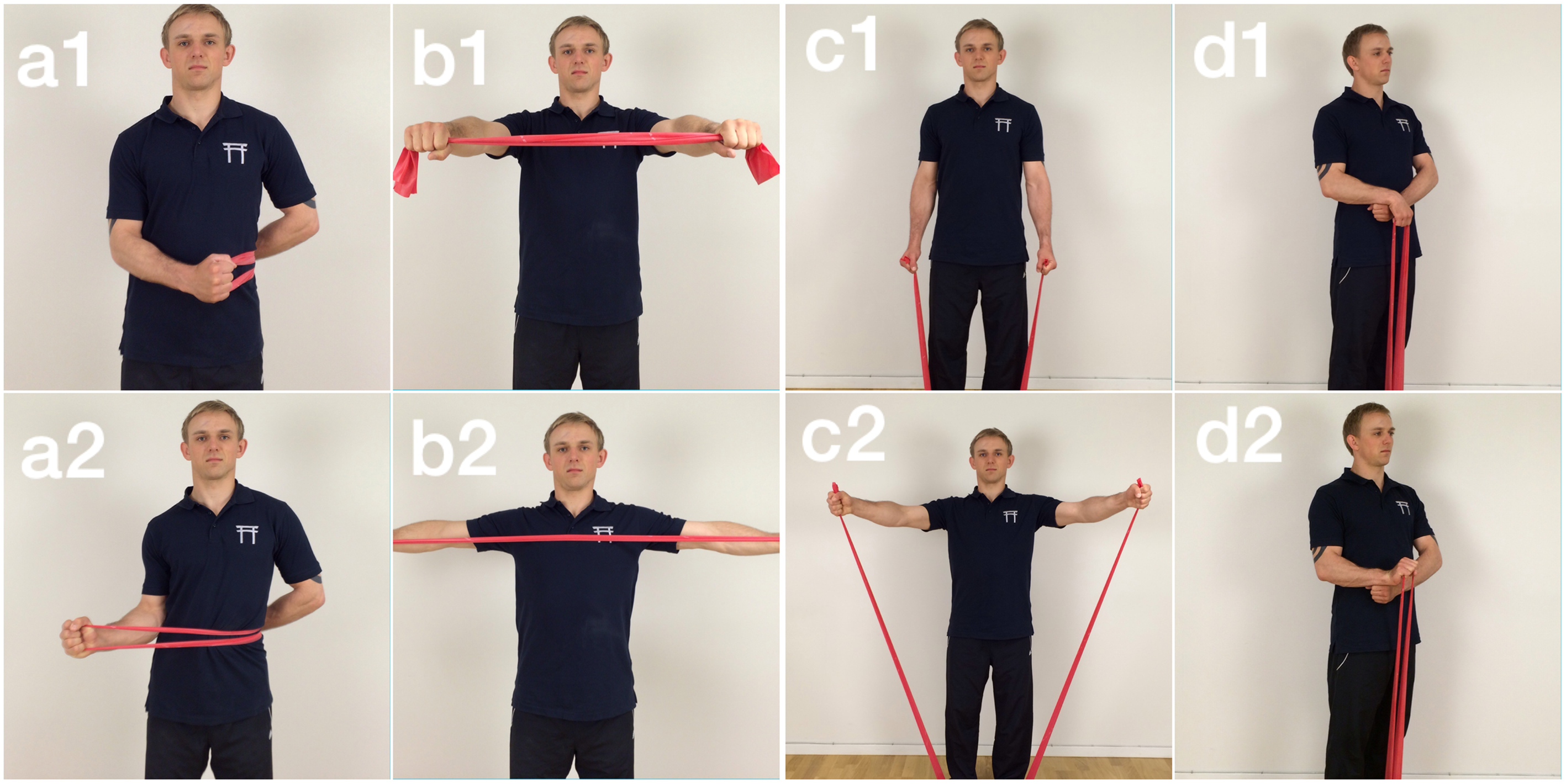

Supplement: Supplementary file 1 — Authors’ original file for figure 1 [file 12891_2014_2398_MOESM1_ESM.tif]

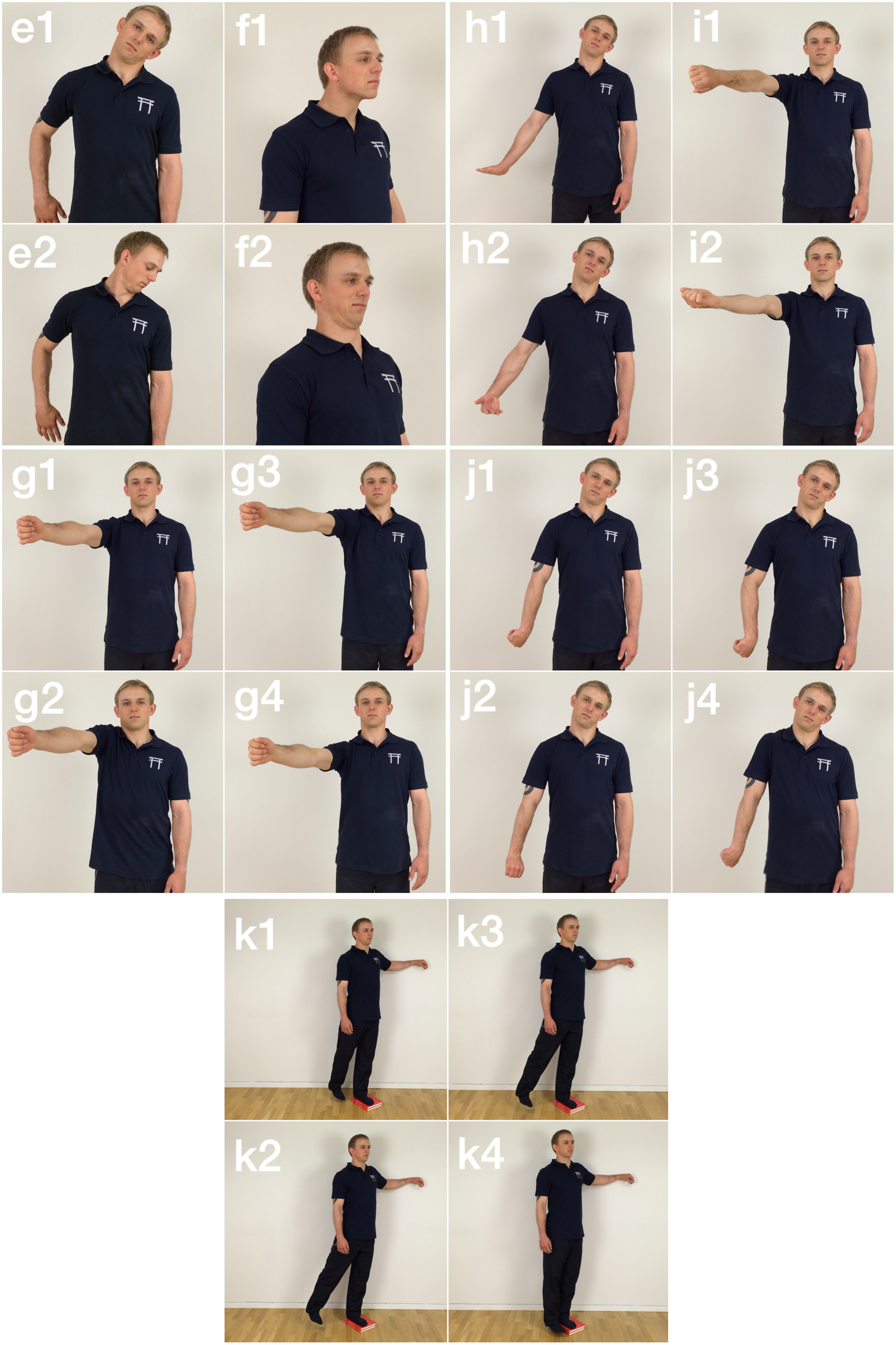

Supplement: Supplementary file 2 — Authors’ original file for figure 2 [file 12891_2014_2398_MOESM2_ESM.tif]

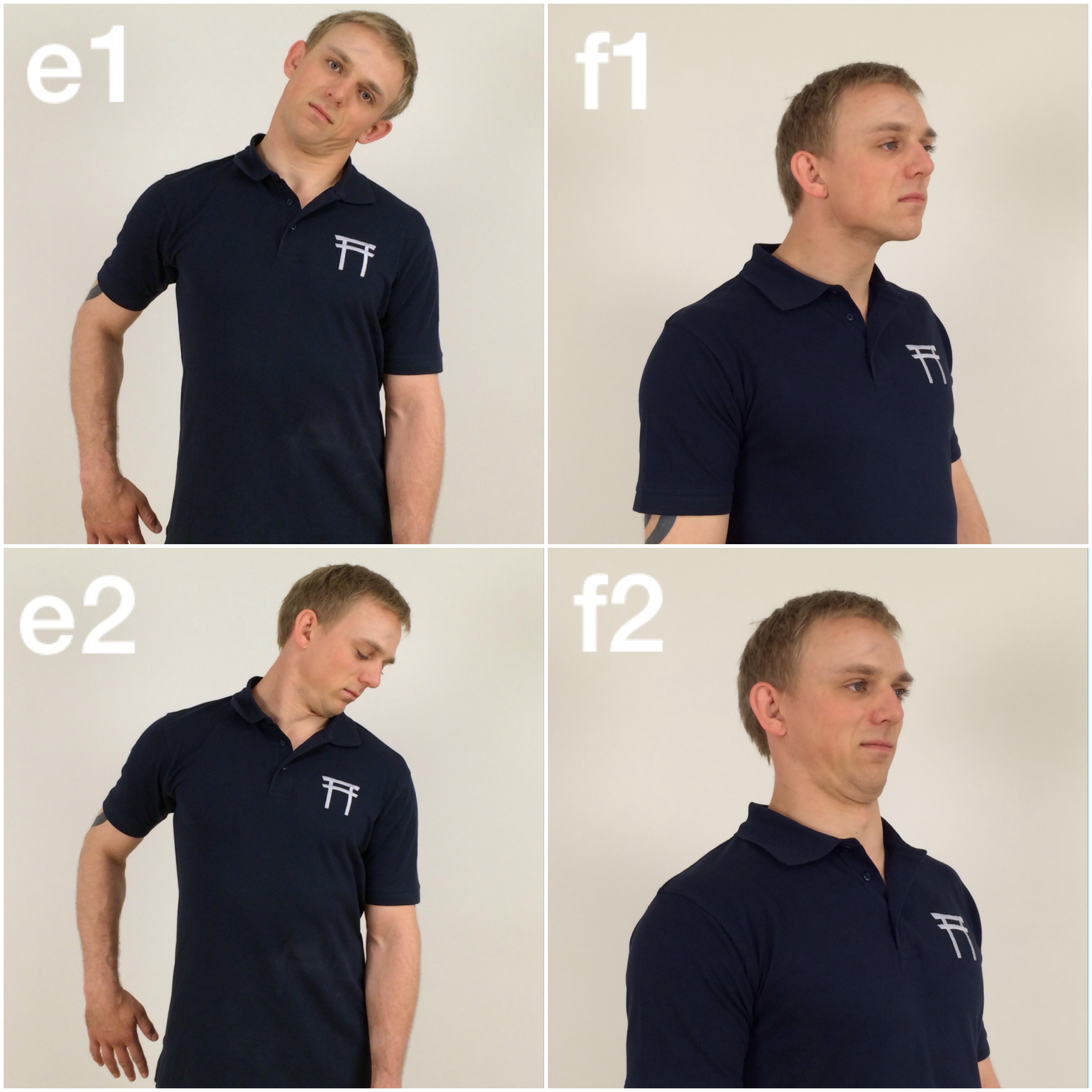

Supplement: Supplementary file 3 — Authors’ original file for figure 3 [file 12891_2014_2398_MOESM3_ESM.jpeg]

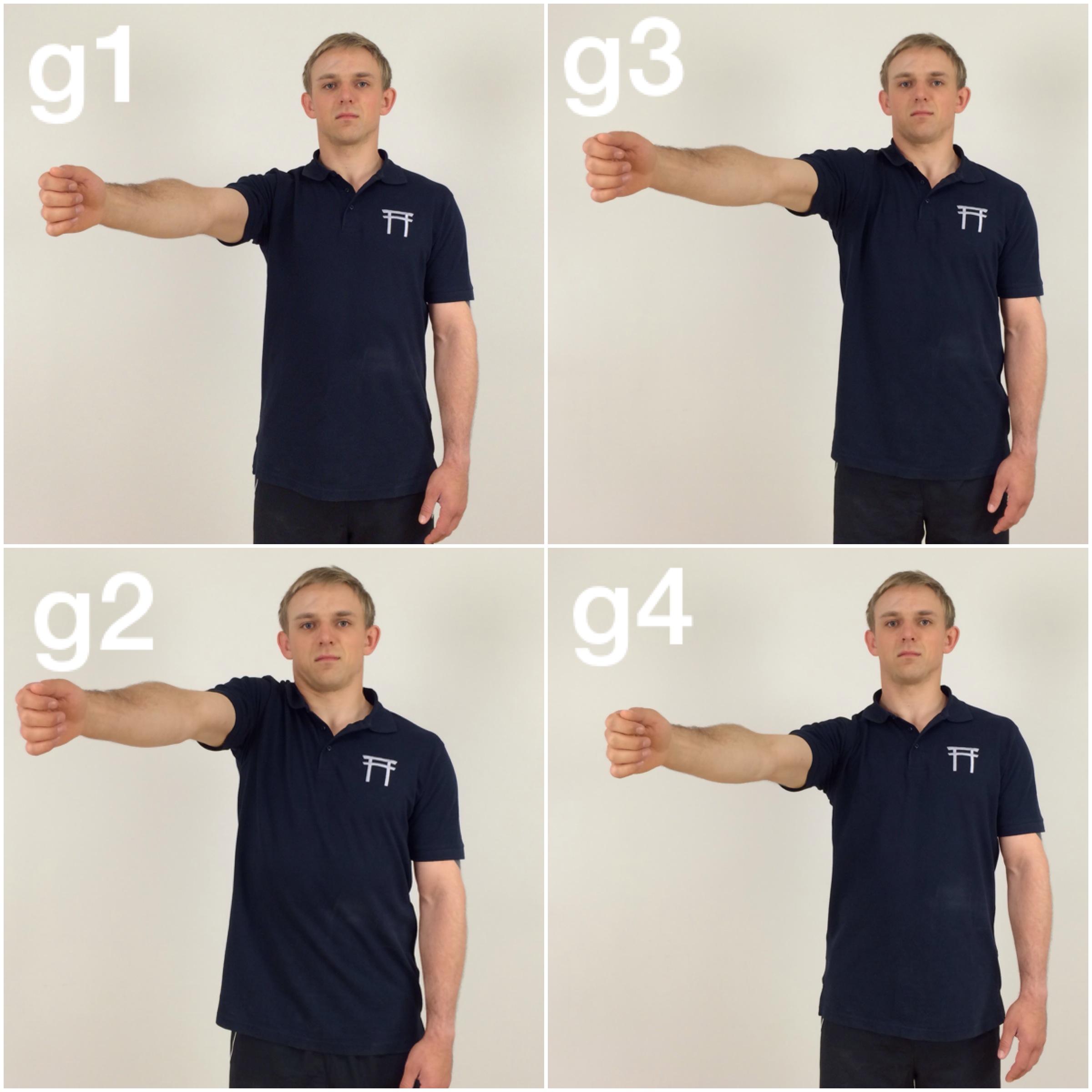

Supplement: Supplementary file 4 — Authors’ original file for figure 4 [file 12891_2014_2398_MOESM4_ESM.jpeg]

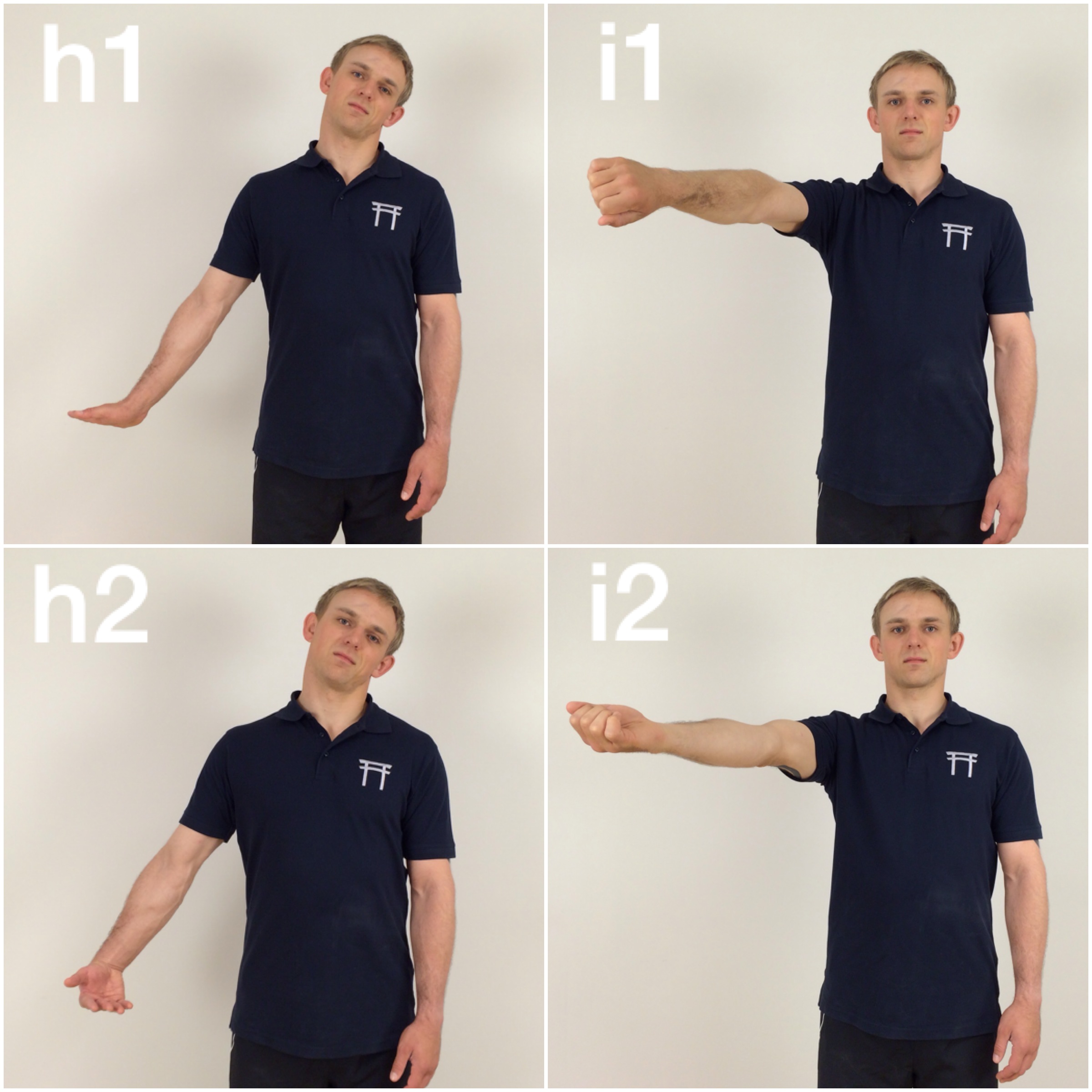

Supplement: Supplementary file 5 — Authors’ original file for figure 5 [file 12891_2014_2398_MOESM5_ESM.jpeg]

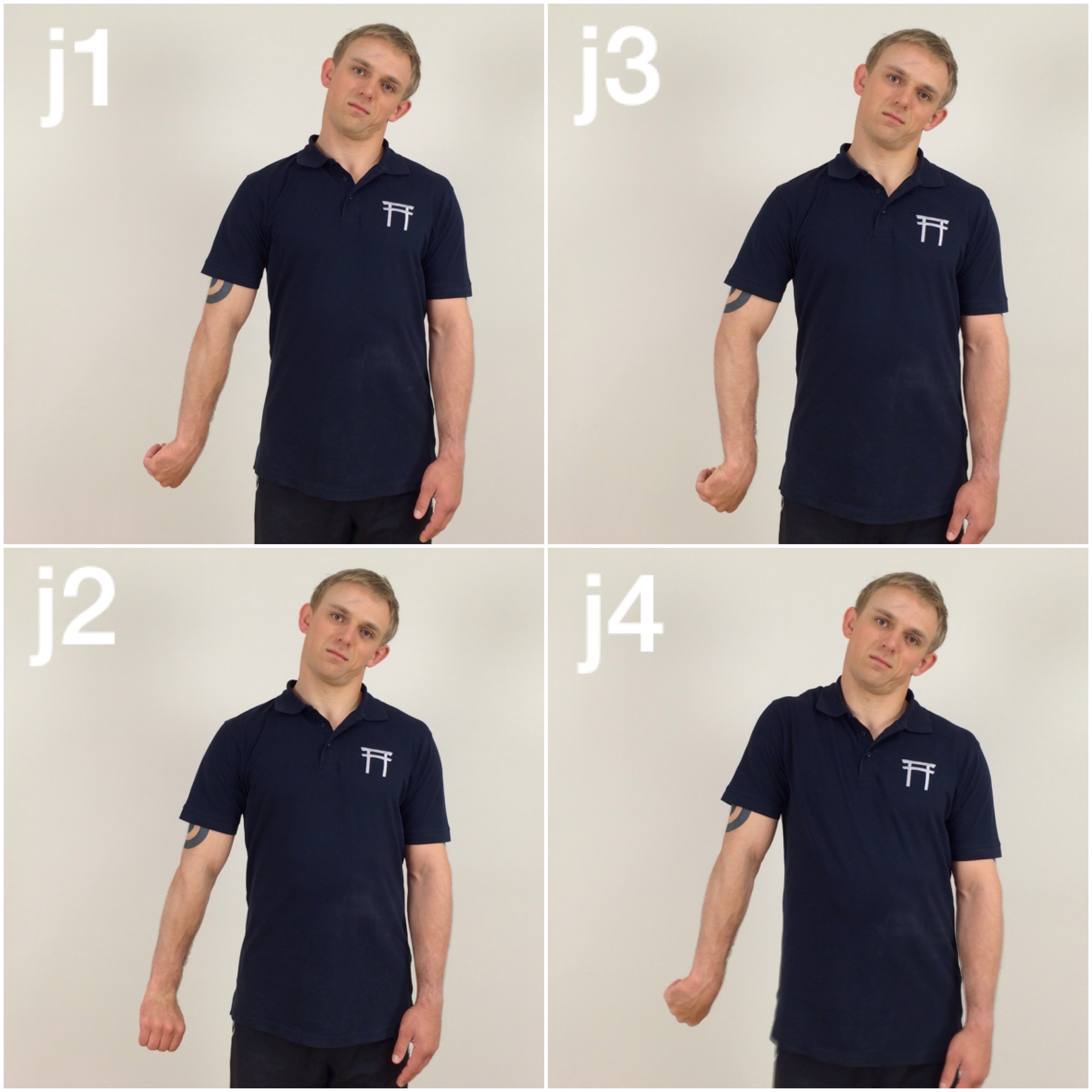

Supplement: Supplementary file 6 — Authors’ original file for figure 6 [file 12891_2014_2398_MOESM6_ESM.jpeg]

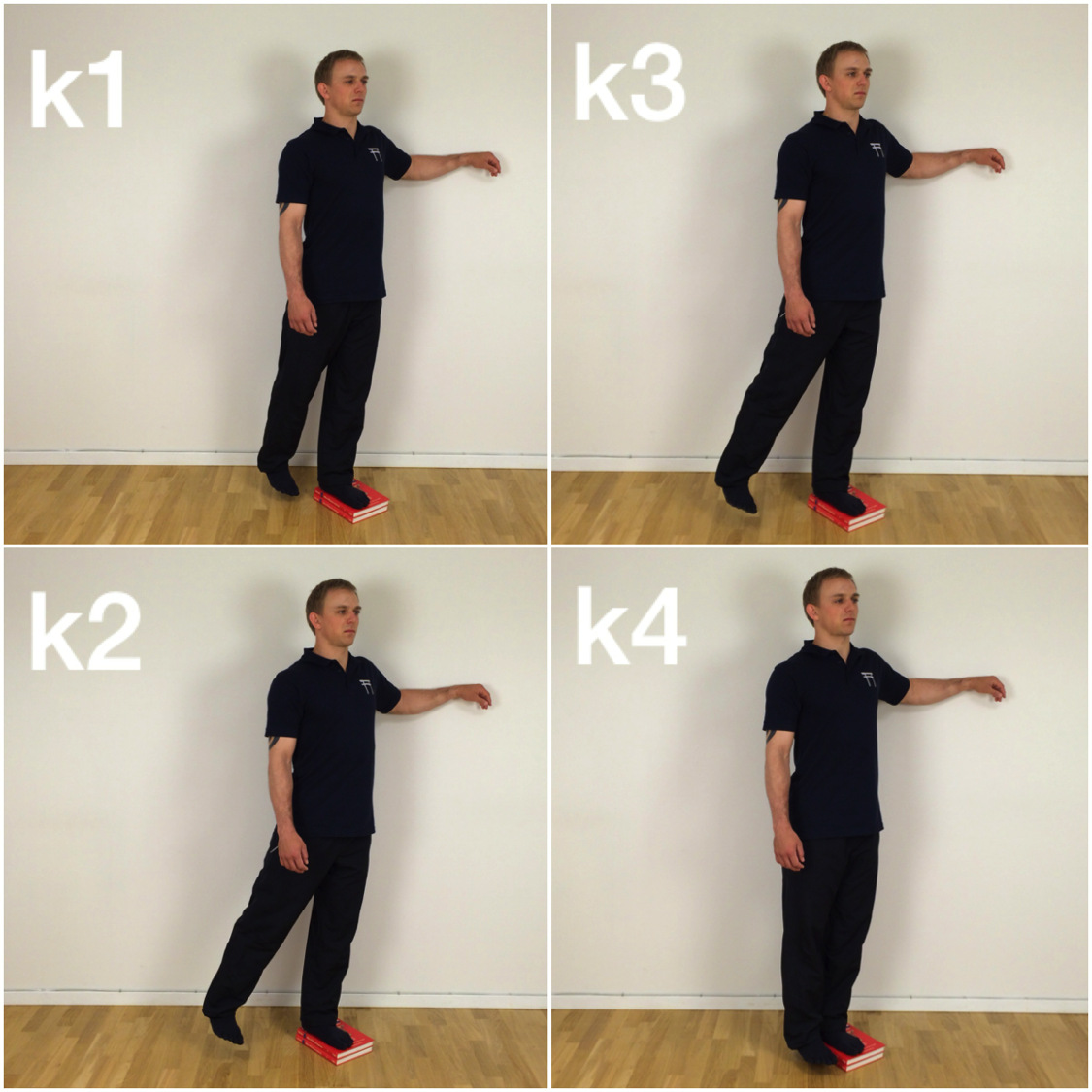

Supplement: Supplementary file 7 — Authors’ original file for figure 7 [file 12891_2014_2398_MOESM7_ESM.png]

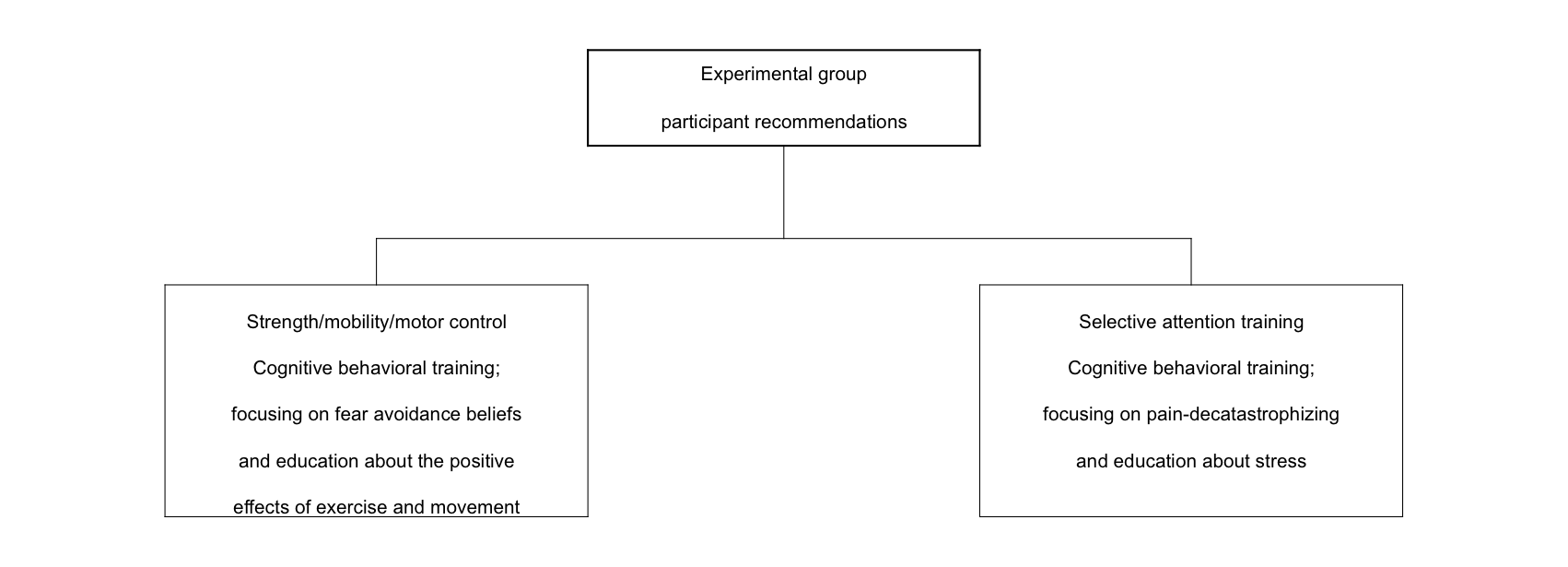

Supplement: Supplementary file 8 — Authors’ original file for figure 8 [file 12891_2014_2398_MOESM8_ESM.png]
